# Error rates in wildlife image classification

**DOI:** 10.1002/ece3.5256

**Published:** 2019-05-22

**Authors:** TJ Gooliaff, Karen E. Hodges

**Affiliations:** ^1^ British Columbia Ministry of Forests, Lands, Natural Resource Operations and Rural Development Penticton British Columbia Canada; ^2^ University of British Columbia Okanagan Kelowna British Columbia Canada

**Keywords:** bobcat, Canada lynx, expert identification, image classification, *Lynx canadensis*, *Lynx rufus*

## Abstract

We address the comments made by Thornton et al. (*Ecology and Evolution*, 2019) in response to our recent article on measuring the agreement among experts in classifying camera images of bobcats and Canada lynx.

We appreciate the response by Thornton, King, Scully, and Murray (2019) to our recent article on measuring the agreement among experts in classifying camera images of bobcats (*Lynx rufus*) and Canada lynx (*Lynx canadensis*; hereafter lynx; Gooliaff & Hodges, 2018). The experiment by Thornton et al. builds upon our work on species classification from camera‐trapping images, but their response minimizes our original findings and fails to recognize our take‐home point: image classification to the species level is often difficult for similar‐looking, sympatric species, and studies with such images should take extra measures to account for this challenge. The inconsistencies in image classification that we uncovered indicate that misclassification rates for similar‐looking species may be high and should be explicitly addressed during study design—points echoed by recent research on images of mice and rats (Burns, Parrott, Rowe, & Phillips, 2017), newts (Austen, Bindemann, Griffiths, & Roberts, 2018), and even for distinguishing between cougars (*Felis concolor*), bobcats, and house cats (*Felis catus*, LaRue, 2018).

Like us, Thornton et al. (2019) measured agreement among a group of classifiers in their classifications of bobcat and lynx images, but they found much higher agreement (Fleiss’ Kappa = 0.87, 95% CI = 0.83–0.93, compared to our Fleiss’ Kappa = 0.64, 95% CI = 0.60–0.68). Even more contrasting, none of the images in their experiment were classified as “unknown” by the classifiers; all of the images were classified as either “bobcat” or “lynx.” This result is strikingly different than the >71% of images in our study that were classified by at least one expert as “unknown.”

We agree with Thornton et al. that these discrepancies in agreement among the image classifiers and the use of “unknown” as a classification are almost certainly explained by methodological differences between our experiments. Specifically, (a) they used 3–5 images for each animal that were all taken by camera traps, whereas we used single images that were taken either from camera traps or from conventional, handheld cameras, (b) they provided specific training and visual aid references on how to distinguish between bobcats and lynx, whereas we provided no prior training, and (c) they asked 56 novice undergraduate students to classify 40 image sequences analyzed at one time, whereas we asked 27 wildlife professionals to classify 300 images divided into six batches of 40–80 images each across three months. Given these significant differences in survey design, it is no surprise that our quantitative results differed. We thus disagree with Thornton et al.'s statement that they used “similar methodology,” nor do we think that their results invalidate our concerns about error rates in image classification.

Although Thornton et al. found higher agreement among their image classifiers, the agreement was still not perfect and the implications of their resulting misclassification rate are ignored by Thornton et al. Unfortunately, they do not state how many image sequences received conflicting classifications, that is, where at least one student classified an image sequence as “bobcat” and at least one student classified it as “lynx”—these data would be valuable as an indicator of misclassification rates per image sequence for their novice classifiers. Thornton et al. found that 3.4% of image sequences (*n* = 77 of 2,240 classifications; 56 students × 40 image sequences = 2,240 individual classifications) were misclassified by the students (assuming the authors’ classifications were correct; it is likely that the error rate would be higher if any of the authors’ classifications were incorrect). This misclassification rate is very similar to the minimum misclassification rate of 3.8% in our study. Thornton et al. did not indicate the distribution of the classification errors, but with 77 errors among 56 students across 40 image sequences, we suspect that most students—and most images—had at least one misclassification. The fact that the recently and intensively trained student classifiers in Thornton et al.'s study had a misclassification rate of at least 3.4% even with 3–5 images of each animal and visual reference guides highlights the difficulty in distinguishing between these similar‐looking species.

Thornton et al. also query how well our study design matched professional practice for image classification; they specifically challenge our use of single images and whether our experts genuinely had the sort of expertise that would be employed in image classification studies. We absolutely agree with Thornton et al. that when multiple images are available, all of them should be used for classification and that the probability of an accurate classification is likely higher; we are pleased to see that many camera‐trapping studies employ settings that will capture multiple images. We note, though, that Thornton et al. were silent on the salient facts that many detections from camera traps still produce only single images, and that studies using citizen‐submitted images taken with conventional cameras may obtain many single images—and such images may be less likely to show characteristic‐defining side profiles of animals than camera traps that are often strategically deployed perpendicular to roads and trails to capture traveling animals. In our original text, we noted that approximately half of the image detections that we solicited from the public consisted of single images (44% of 837 detections from camera traps and 52% of 748 detections from conventional cameras). Our experimental design thus enables us to speak directly about the difficulty in classifying a large proportion of the data collected in camera‐trapping and public‐solicitation studies.

Thornton et al. also challenge whether the experts in our study offered an appropriate sample, especially since some of them had worked extensively with one species rather than with both. We note simply that all of the experts we sampled had conducted research or management sampling for at least one of the species, and they were in professional positions such that members of the public (or even others in their departments) would come to them with images for classification. We agree with Kosmala, Wiggins, Swanson, and Simmons (2016) and Austen et al. (2018) that people vary in what they recognize as expertise, but we believe that the positions and experience held by the people we sampled would meet most such definitions.

We also note that Thornton et al. mischaracterized our statements about experts and nonexperts; Thornton et al. claim that we “conclude[d] that misclassification rates would be even higher when classified by nonexperts despite not having tested this assertion explicitly.” In fact, we were careful to signal that we were making a prediction: we stated “Misclassification rates would also likely be higher when images are classified by nonexperts, such as volunteers and crowdsourcing… we strongly suggest caution when classifying images for species with similar sympatrics.” Our caution is supported by LaRue (2018), who runs the Twitter‐based quiz #CougarOrNot in which nonexpert individuals can vote yes (it is a cougar) or no (not a cougar) on single images (of cougars, house cats, bobcats, other predators, etc.); she finds that individual images are accurately classified by <17% to 90% of respondents, thus yielding high error rates even for such dissimilar animals. In the context of the experiment that Thornton et al. provided, which they claim is evidence for skill among nonexperts, we note that they did not test single images and their classroom‐based explicit training is not the situation that we addressed.

The experiment by Thornton et al. strongly suggests that training and providing classifiers with detailed visual aids improves image classification, echoing results from Kosmala et al. (2016) and Sharma, Colucci‐Gray, Siddharthan, Comont, and Wal (2019). We still do predict that inexperienced and untrained undergraduate students would have poorer agreement compared to the experts used in our study if both groups examined the same images. Thus, training and detailed visual aids appear to be useful in image classification studies that use novice classifiers, which is an important finding of Thornton et al. We chose to not train the classifiers in our study because we wanted to survey classification agreement for people who would be expected to be able to distinguish between the two species as part of their current professional practice.

Thornton et al. also challenge our recommendation that five experts be consulted for images that contain bobcats or lynx. We retain this recommendation because our results did show that for single images classified by current wildlife professionals, error rates in image classification were high, as was the use of “unknown” as a classification. Consulting five experts would achieve a high probability that the majority classification among those classifiers would not change with the asking of additional experts. Given that their results—from 3–5 images per animal—still had an error rate of at least 3.4%, we are not willing to recommend using classifications drawn from fewer observers.

We close by reiterating the importance of the study design and the downstream implications of erroneous image classifications. Even the 3.4% misclassification rate from Thornton et al. would add up to many misclassifications in studies with many detections. If a study occurs where both lynx and bobcats are common, misclassifications are less likely to lead to serious mistakes in inference or to induce poor management decisions. In contrast, in studies like our other work (Gooliaff, Weir, & Hodges, 2018) where part of the purpose is to determine species’ distributions, misclassification of images from the range edge of a species could lead to flawed results. In our case, had we relied only on images, misclassifying even a few of the northernmost bobcat classifications would have resulted in a distribution map that ended a couple hundred kilometers south of the final distribution map (our work included trapped individuals and other data sources). For lynx in northern Washington, where populations are federally threatened and state‐listed as Endangered, misclassifications of lynx as bobcats could underestimate the distribution or occupancy of lynx, whereas misclassifications of bobcats as lynx could result in a false sense of security about the lynx population.

Thus, we are glad to see the results from Thornton et al. (2019), as they show that recently trained novice classifiers working from multiple images can obtain reasonably high agreement with each other, although individual classifications still have a sizeable error rate. Their work reinforces our main points that (a) studying error rates in image classification is important, (b) researchers should document how images were classified and what steps were taken to reduce or manage misclassifications (whether via training or consultation of many experts or novices), and (c) the research or management context in which the work is undertaken will affect how important errors are for subsequent inference and management actions. These ideas have wide backing in fields as disparate as ecology (e.g., camera traps), medicine (e.g., screening and diagnostic imagery; Welch, Schwartz, & Woloshin, 2011), and forensics (e.g., bite marks, tire prints, and fingerprints, Saks & Faigman, 2008), as all of these fields have error rates in classification that are affected by image attributes and by the classifying individuals and their training. The central issues are to identify, manage, and work to reduce background error rates. We look forward to future research in ecology on image classification as this noninvasive survey technique becomes more important, especially for work on endangered wildlife species.

## CONFLICT OF INTEREST

None declared.
